# Informing the Historical Record of Experimental Nonhuman Primate Infections with Ebola Virus: Genomic Characterization of USAMRIID Ebola Virus/H.sapiens-tc/COD/1995/Kikwit-9510621 Challenge Stock “R4368” and Its Replacement “R4415”

**DOI:** 10.1371/journal.pone.0150919

**Published:** 2016-03-22

**Authors:** Jeffrey R. Kugelman, Cynthia A. Rossi, Michael R. Wiley, Jason T. Ladner, Elyse R. Nagle, Bradley P. Pfeffer, Karla Garcia, Karla Prieto, Jiro Wada, Jens H. Kuhn, Gustavo Palacios

**Affiliations:** 1 United States Army Medical Research Institute of Infectious Diseases (USAMRIID), Fort Detrick, Frederick, Maryland, United States of America; 2 Integrated Research Facility at Fort Detrick (IRF-Frederick), National Institute of Allergy and Infectious Diseases, National Institutes of Health, Fort Detrick, Frederick, Maryland, United States of America; University of Texas Medical Branch, UNITED STATES

## Abstract

The creation of licensed medical countermeasures against Select Agents such as Ebola virus (EBOV) is critically dependent on the use of standardized reagents, assays, and animal models. We performed full genome reconstruction, population genomics, contaminant analysis, and characterization of the glycoprotein gene editing site of historical United States Army Medical Research Institute of Infectious Diseases (USAMRIID) nonhuman-primate challenge stock Ebola virus Kikwit “R4368” and its 2014 replacement “R4415.” We also provide characterization of the master stock used to create “R4415.” The obtained data are essential to understanding the quality of the seed stock reagents used in pivotal animal studies that have been used to inform medical countermeasure development. Furthermore, these data might add to the understanding of the influence of EBOV variant populations on pathogenesis and disease outcome and inform attempts to avoid the evolution of EBOV escape mutants in response to current therapeutics. Finally, as the primary challenge stocks have changed over time, these data will provide a baseline for understanding and correlating past and future animal study results.

## Introduction

Ebola virus disease (EVD) is a frequently lethal human viral hemorrhagic fever caused by four distinct ebolaviruses (Bundibugyo virus, Ebola virus, Sudan virus, and Taï Forest virus). EVD occurs sporadically and usually affects no more than several hundred people during an outbreak (reviewed in [1]). In 2014, Ebola virus (EBOV) was identified as the etiological agent of an unprecedented EVD outbreak that started in Western Africa in late 2013 and has since caused 28,637 cases and 11,315 deaths (as of January 3, 2016) [2].

Taxonomically, EBOV is the only member of the species *Zaire ebolavirus* in the genus *Ebolavirus* (*Mononegavirales*: *Filoviridae*) [3]. The EBOV genome is rather conserved over time and geographic distances, which may be due to genetic bottlenecks in the yet-unidentified host reservoir [1, 4]. For instance, the EBOV variant that caused the 2013–present outbreak in Western Africa (Makona [5]) differs from those that caused EVD outbreaks in Zaire in 1976 (Yambuku [6, 7]) and Zaire’s successor country Democratic Republic of the Congo in 1995 (Kikwit [8]) by less than 3% over the entire ≈19 kb genome [9].

The development, evaluation, and final licensure of medical countermeasures (MCMs) against EVD in the US is critically dependent on standardized animal models of filovirus infection and standardized assays and reagents [10–12], including well-characterized virus stocks. Ebola virus/H.sapiens-tc/COD/1976/Yambuku-Mayinga (EBOV/Yam-May) is the best characterized EBOV isolate and has been used for the majority of *in vitro* experiments [1, 7, 13]. Ebola virus/H.sapiens-tc/COD/1995/Kikwit-9510621 (EBOV/Kik-9510621), on the other hand, has become the most used EBOV isolate for animal, and in particular nonhuman primate (NHP), experimentation in the United States (US) [1, 13, 14]. Unfortunately, this isolate has been passaged/maintained by different procedures in different locations and even within the same institutes. These variable procedures result in NHP challenge stocks of different quality and possibly in different experimental outcomes upon use. In addition, only one of these NHP challenge stocks has been genomically characterized to assess potential mutations relative to the published consensus genome sequence [14].

All ebolaviruses make use of co-transcriptional editing of their glycoprotein-encoding *GP* genes to access three partially overlapping open reading frames (ORF) [15, 16]. Editing occurs at a distinct editing site in the *GP* gene that typically consists of seven consecutive uridylyls (7U). Read-through results in the transcription of a cognate 7 adenylyl mRNA (7A mRNA) and thereby in the expression of pre-sGP, a protein precursor that is proteolytically processed to a secreted homodimeric glycoprotein (sGP) and a probably monomeric secreted peptide (Δ-peptide). Stuttering of the ebolavirus RNA-dependent RNA polymerase (L) at the editing site results in transcription of mRNAs that contain various numbers of adenylyls. The majority of non-7A mRNAs contain eight adenylyls, leading to the expression of the homotrimeric ebolavirus spike glycoprotein GP_1,2_, or six or nine adenylyls, leading to expression of the small soluble glycoprotein (ssGP) [15–20]. The functions of sGP, ssGP, and Δ-peptide are largely unknown but a role for sGP in immune evasion has been described [21]. Editing appears to be tightly controlled, with the ratio of proteins expressed from a 7U virus of roughly sGP:GP_1,2_:ssGP = 75%:25%:5% [17, 22].

EBOV, Sudan virus (SUDV), and possibly other ebolaviruses adapt to different *in vitro* and *in vivo* environments [14, 23–25]. These adaptations include changes in the EBOV and SUDV *GP* gene editing sites. For instance, during serial passage of a 7U EBOV/Yam-May isolate in Vero E6 cells, a viral population evolved that predominantly contained an 8U editing site. The opposite occurs *in vivo*: 8U EBOV/Yam-May populations converted to 7U populations in guinea pigs [10]. These changes may be related to selective advantages linked to the controlled expression of GP_1,2_ and/or sGP. Preliminary evaluations indicate that a 7U→8U mutation in EBOV does not alter pathogenesis in guinea pigs or nonhuman primates [25]. However, the observations described above indicate that there may be selective advantages associated with the ratio of expressed sGP:GP_1,2_ in different environments (8U-containing viruses predominantly express GP_1,2_ rather than sGP). In addition, a recent study reported subtle, but significant, differences in disease course and severity between nonhuman primates exposed to 7U or 8U virus stock variants [26]. All of these findings underscore the importance of fully characterizing viral stocks used in MCM development.

Here, we report the coding-complete genome sequence (see [27] for sequencing terminology), population characterization, and contaminant analysis of an EBOV/Kik-9510621 challenge stock “R4368” (passage 4, 8U) that was used in the past for NHP studies at the United States Army Medical Research Institute of Infectious Diseases (USAMRIID) at Fort Detrick, Maryland. We further report the complete genome of sequence at the same level of characterization of challenge stock “R4415” (passage 3, 7U), which replaced “R4368” (passage 4, 8U) in 2014, and the master stock used to establish this new stock (“R4414” (passage 2, 7U)).

### History of USAMRIID EBOV/Kik-9510621 challenge stocks

A clinical specimen (designated Centers for Disease Control and Prevention Special Pathogens Branch Log [CDC SPBLOG] 9510621) was obtained from an EBOV-infected 65-year old female patient during an EVD outbreak that occurred in 1995 around Kikwit, Zaire (today Democratic Republic of the Congo, COD). The patient’s disease onset was recorded as April 29, 1995. She was hospitalized on May 1, 1995, and died on May 5, 1995. The clinical specimen, most likely plasma or serum, was obtained from the patient on May 4, 1995. How the patient became infected and what medical care she may have received is unclear. Unfortunately, chain-of-custody records that further detail the origin of 9510621 or its shipment to the CDC are not available anymore, and EBOV titration was not attempted from this specimen prior to cell-culture passage.

**Fig 1:**
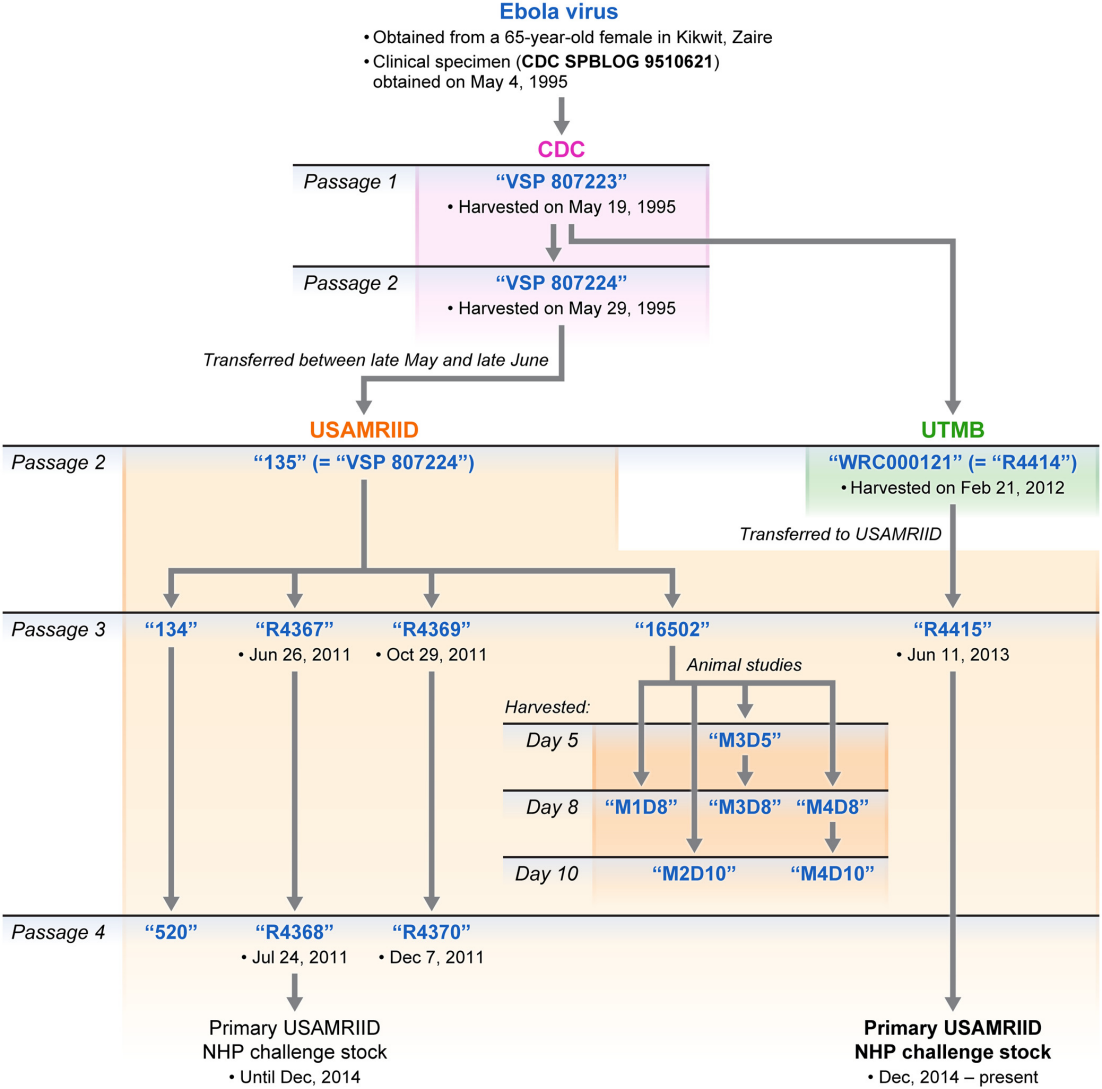
History of Ebola virus variant Kikwit (isolate 9510621) challenge stocks used at the United States Army Medical Research Institute of Infectious Diseases (USAMRIID), CDC, Centers for Disease Control and Prevention; NHP, nonhuman primate; SPBLOG, Special Pathogens Branch Log; UTMB, University of Texas Medical Branch; VSP, virus seed pool.

The first passage of EBOV/Kik-9510621, designated “virus seed pool (VSP) 807223” (Fig 1), was conducted at the CDC using grivet (*Chlorocebus aethiops*) Vero E6 cells (ATCC #CRL-1586). The multiplicity of infection (MOI) used for this passage is unknown. Virus was harvested after a 6-day incubation period on May 19, 1995, but the method of harvest (initial freeze/thaw, clarification by centrifugation etc.) is not indicated in the available records. A second passage of virus, was conducted at the CDC, again using Vero E6 cells, and resulted in VSP 807224 (MOI unknown). Virus was harvested after an 8-day incubation, using a “freeze/thaw” method, on May 29, 1995. A titer of 3.2E+06 was determined for “VSP 807224” in January 2008 using the TCID_50_/Reed-Munch viral titration method. “VSP 807224” was transferred from CDC between late May and late June 1995 to USAMRIID.

At USAMRIID, “VSP 807224” was stored frozen at -70°C and designated there as “135” (passage 2). After an additional passage in Vero E6 cells, the virus, now designated “134” (passage 3), was sequenced by Chain *et al*. using classical dideoxynucleotide sequencing. “134” (passage 3) is cell culture-adapted, consisting predominantly of viruses with an 8 uridylyl (8U) glycoprotein (*GP*) gene editing site. A coding-complete genome for “134” was deposited on July 24, 2003, in GenBank under accession #AY354458. “135” (passage 2) was amplified at USAMRIID at least three more times (and in the process was depleted), leading to three separate NHP challenge stocks: “R4367” (passage 3, MOI 0.001), “R4369” (passage 3, MOI 0.01), and “16502” (passage 3, MOI not available). The genomic characterization of stock “16502” (passage 3) and its evolution in a nonhuman primate study over several days was previously described [14] (Fig 1). “R4367” (passage 3) was passaged on Vero E6 cells at an MOI of 0.001, collected, and clarified on day 5 (June 26, 2011), and then passaged one more time at an MOI of 0.01 on Vero E6 cells to yield “R4368” (passage 4). “R4368” (passage 4) was collected and clarified on day 4 (July 24, 2011). “R4369” (passage 3) was passaged on Vero E6 cells at an MOI of 0.01 and collected and clarified on day 4 (October 29, 2011). Titers of “R4367” (3.31E+06 plaque-forming units (pfu)/ml), “R4368” (4.56E+06 pfu/ml), and “R4369” (7.01E+06 pfu/ml) were established using an agarose-based plaque assay [21]. Each of these three preparations was harvested after 2–3+ cytopathic effect (CPE) had developed.

Independently at UTMB, “VSP 807223” was passaged one time on Vero E6 cells, harvested after a 10-day incubation period on February 21, 2012, and stored in 1.0-ml aliquots as “WRC000121” (passage 2; MOI unknown). A titer of 1.8E+07 pfu/ml was determined (titration method unknown). On May 1, 2012, UTMB transferred “WRC000121” to USAMRIID, where the virus was stored at -70°C and designated as “R4414” (passage 2). This material was passaged at an MOI of 0.001 on Vero E6 cells to prepare NHP challenge stock “R4415” (passage 3), which was harvested on June 11, 2013, after a 12-day incubation period (time to develop 2–3+ CPE) with a titer of 1.31E+06 pfu/ml determined by agarose-based plaque assay [21]. The primary USAMRIID NHP challenge stock “R4368” (passage 4, 8U) was replaced by “R4415” (passage 3, 7U) in December of 2014 and is now USAMRIID’s primary NHP challenge stock.

## Materials and Methods

Vials (1 ml) of EBOV/Kik-9510621 challenge stocks were stored and maintained at USAMRIID at -70°C. “R4368” (passage 4) was stored since July 24, 2011, and thawed on March 2, 2012; “R4414” (passage 2) and “R4415” (passage 3) were stored since June 11, 2013, and were thawed in January 2014. An aliquot of 100 μl of each thawed virus stock was placed in 3:1 TRIzol (Life Technologies, Carlsbad, CA). Nucleic acids were isolated from TRIzol-treated material, and genome sequence was determined on Illumina technology (MiSeq or HiSeq) with EBOV-specific oligonucleotides following sample preparation performed as described in [14]. The consensus genomes were generated via reference alignment to EBOV/Kik-9510621 challenge stock “134” (passage 3) (GenBank #AY354458) using SeqMan nGen (DNASTAR). The resulting “R4368” (passage 4), “R4414” (passage 2), and “R4415” (passage 3) sequences were deposited in GenBank under accession numbers KT582109, KT762961, and KT762962, respectively. The population genetics assembly files and sequence-independent, single-primer amplification (SISPA) raw sequence used for contaminant analysis are available in BioSample database under SRS1037973, SRS1041428, and SRS1041442.

For rapid amplification of cDNA ends (RACE), “R4415” (passage 3) RNAs were extracted with Zymo Direct-Zol (Zymo Research Corporation, Irvine, CA) from cell-culture supernatant in TRIzol according to the manufacturers' instructions. SMARter RACE 5’/3’ kit (Clontech Laboratories, Inc., Mountain View, CA) was used to amplify both 5' and 3' untranslated regions (UTRs) of the virus genome from the extracted RNAs. The kit's two-stage nested PCR protocol was found to be optimal. The gene-specific primers for each RACE experiment are as follows 5’ RACE (outer primer): ATTACCAGAGTTGATTAGTGTG; 5’ RACE (inner primer): TTAAATAACGAAAGGAGTC; 3’ RACE (outer primer): TGAATCTCCAATCCTCTAAGTA; 3’ RACE (inner primer): AAGGGATTTTCAACTGAGCACACT. Amplification primer removal, duplicate removal, low average quality exclusion (≤ Q30), and quality trimming was performed. Viral assemblies were completed in DNAStar Lasergene nGen (Madison, WI) with ≈4×10^5^ reads. Only single nucleotide polymorphism (SNPs) present in the population above the 2% threshold are presented in this report (however, the alignment files are provided in SRA if a less conservative approach is desired). Considering this threshold, a target depth of 200 requires an SNP to have ≥ 4 supporting reads prior to being called a SNP. The depths are reported in the tables presented in this text for all samples (below 200 depth, calls should be viewed with increasing skepticism). A consensus change is defined here as a change relative to the published sequence for EBOV/Kik-9510621 “134” (GenBank accession # AY354458) present in ≥50% of the population. Below that threshold, SNPs are considered subclonal substitutions and part of a minority subpopulation of the virus.

### Ethics Statement

Research has been reviewed for compliance with dual-use guidelines and approved for publication by the USAMRIID Institute Biosafety Committee (IBC) and the Operational Security office.

## Results

Compared to EBOV/Kik-9510621 “134” (passage 3), “R4368” (passage 4) acquired only one consensus-level substitution (nucleotide position 7,327). Six intrahost single nucleotide variants (iSNVs) (≥2% of the population) were detected and are reported in Table 1. Compared to EBOV/Kik-9510621 “134” (passage 3), “R4414” (passage 2) acquired three consensus-level substitutions (nucleotide positions 6,179, 7,327, 10,833). Eleven iSNVs (≥2% of the population) were detected and are reported in Table 2. An additional passage-acquired mutation was observed in the challenge stock “R4415” (passage 3) (nucleotide position 7,669) when compared to “R4414” (passage 2). Thirteen iSNVs (≥2% of the population) were detected and are reported in Table 3. No significant contaminants were detected. The genotype ratios at the GP editing site for all three stocks are detailed in Table 4. As expected based on the “134” (passage 3) progenitor sequence, “R4368” (passage 4) predominantly encodes the 8U genotype (85.0%). “R4414” (passage 2) and “R4415” (passage 3) predominantly encode the 7U genotype.

**Table 1:** Intrahost single nucleotide variants (iSNVs) of EBOV/Kik-9510621 “R4368” (passage 4) comprising ≥2% of total population as compared to “134” (GenBank #AY354458).

Reference position (“134” (passage 3))	Reference base	SNP base	SNP %	Codon	Gene	Depth
5,878	T	g	2.84			1374
6,139	C	t	2.07	P (CCA) @34 L (CtA)	*GP*	1595
6,179	G	t	8.57	E (GAG) @47 D (GAt)	*GP*	1541
7,298	T	c	3.98	Synonymous	*GP*	804
7,327	C	T	99.61	P (CCA) @430 L (CTA)	*GP*	507
10,833	G	a	2.35	R (AGA) @163 K (AaA)	*VP24*	1533
16,365	A	g	2.76	Q (CAG) @1595 R (CgG)	*L*	1560

SNP, single nucleotide polymorphism.

**Table 2:** Intrahost single nucleotide variants (iSNVs) of EBOV/Kik-9510621 “R4414” (passage 2) comprising ≥2% of total population as compared to “134” (GenBank #AY354458).

Reference position (“134” (passage 3))	Reference base	SNP base	SNP %	Codon	Gene	Depth
1,401	G	a	2.08	G:GGT @ 311 → D:GaT	*NP*	1,008
5,830	T	a	2.18		*VP40*	1,789
6,179	G	t	100.00	E:GAG @ 47 → D:GAt	*GP*	2,887
6,231	T	c	3.97	S:TCA @ 65 → P:cCA	*GP*	3,956
6,384	C	a	4.33	P:CCT @ 116 → T:aCT	*GP*	5,264
7,327	C	t	99.90	P:CCA @ 430 → L:CtA	*GP*	995
7,669	C	t	33.00	T:ACA @ 544 → I:AtA	*GP*	912
10,344	C	a	4.60		*VP24*	5,108
10,833	G	a	99.60	R:AGA @ 163 → K:AaA	*VP24*	3,422
11,283	A	c	2.89		*VP24*	3,361
11,498	G	a	6.05			1,123
12,065	G	a	4.21	G:GGT @ 162 → S:aGT	*L*	4,798
12,153	G	t	5.38	W:TGG @ 191 → L:TtG	*L*	5,246
14,184	C	t	2.13	S:TCG @ 868 → L:TtG	*L*	1,880

SNP, single nucleotide polymorphism.

**Table 3:** Intrahost single nucleotide variants (iSNVs) of EBOV/Kik-9510621 “R4415” (passage 3) comprising ≥2% of total population as compared to “134” (GenBank #AY354458).

Reference position (“134” (passage 3))	Reference base	SNP base	SNP %	Codon	Gene	Depth
520	T	c	10.60	S:TCT @ 17 →→ S:TCc	*NP*	4,330
530	T	c	10.15	Y:TAC @ 21 → H:cAC	*NP*	4,403
542	T	c	9.84	L:TTG @ 25 → L:cTG	*NP*	4,483
606	T	c	10.70	V:GTA @ 46 → A:GcA	*NP*	4,831
1,274	A	g	7.83	R:AGG @ 269 → G:gGG	*NP*	5,62
5,830	T	c	3.20		*VP40*	2,871
6,179	G	t	99.90	E:GAG @ 47 → D:GAt	*GP*	8,337
6,384	C	a	3.4	P:CCT @ 116 → T:aCT	*GP*	11,383
7,327	C	t	99.940	P:CCA @ 430 → L:CtA	*GP*	1,048
7,669	C	t	98.70	T:ACA @ 544 → I:AtA	*GP*	1,486
10,344	C	a	4.20		*VP24*	9,735
10,833	G	a	100.00	R:AGA @ 163 → K:AaA	*VP24*	4,642
11,283	A	c	3.91		*VP24*	1,844
11,498	G	a	5.15			466
12153	G	a	4.95	W:TGG @ 191 →.:TaG	*L*	4,910
13994	C	a	4.65	Q:CAA @ 805 → K:aAA	*L*	7,614
16247	T	c	2.19	S:TCA @ 1556 → P:cCA	*L*	2,838

SNP, single nucleotide polymorphism.

**Table 4:** *GP* gene editing site composition in EBOV/Kik-9510621 NHP challenge stocks.

	6U/9U (ssGP phenotype)	7U (sGP phenotype)	8U (GP_1,2_ phenotype)
**“R4368” (passage 4)**	3.8% (373)	11.2% (1,090)	85.0% (8,300)
**“R4414” (passage 2)**	0.6% (149)	97.5% (23222)	1.8% (439)
**“R4415” (passage 3)**	0.3% (40)	88.8% (11231)	10.9% (1378)

GP, glycoprotein; sGP, soluble glycoprotein; ssGP, small secreted glycoprotein; 7–9U, 7–9 uridylyl glycoprotein (*GP*) gene editing site.

## Discussion

We have provided here a concise report on the history and genomic characterization of the USAMRIID Ebola virus/H.sapiens-tc/COD/1995/Kikwit-9510621 NHP challenge stocks “R4368” (passage 4) and “R4415” (passage 3), as well as the “R4415” (passage 3) predecessor, “R4414 (passage 2).” “R4368” (passage 4) was used between July 2011 and December 2014 in both *in vitro* and *in vivo* Ebola virus experimentation, including pathogenesis studies and candidate medical countermeasure evaluation. “R4415” (passage 3) replaced “R4368” (passage 4) in December 2014 and has been used for most pathogenesis and medical countermeasure evaluation research since then. This work provides a framework for genomic comparison between past experiments as challenge stocks are replaced to address propagation issues and depletion. Characterization of these NHP challenge stocks was completed to the level of “Coding Complete” plus population-level characterization in the case of “R4368” (passage 4) and “Finished” in the case of “R4415” (passage 3) [27]. This characterization includes genome reconstruction (excluding determination of the 3’ and 5’ UTRs in the case of “R4414” (passage 2) and “R4415” (passage 3), characterization of intrahost variants (iSNVs), and determination of absence of contaminants. Studies to determine the role of the identified iSNVs in interactions with the host (e.g., in the immune response) are being considered for expansion of this body of work. This level of characterization is crucial for studies evaluating the possibility of EBOV escape from candidate therapeutics or vaccines, as minority variants can play an important phenotypic role in viral escape [28].
